# Efficacy of Action Observation Therapy on Cognitive Function in Stroke: A Systematic Review and Meta‐Analysis

**DOI:** 10.1002/brb3.70474

**Published:** 2025-04-21

**Authors:** Xuewei Guan, Meijuan Lan, Leiwen Tang, Hongyan Yang, Yuanyuan Chen, Lan Ge, Yumei Zhong

**Affiliations:** ^1^ Nursing Department The Second Affiliated Hospital of Zhejiang University School of Medicine Hangzhou China

**Keywords:** Action Observation Therapy, cognitive function, meta‐analysis, stroke

## Abstract

**Introduction**: Action Observation Therapy (AOT) is a rehabilitation method believed to activate the mirror neuron system, which may contribute to cognitive recovery. Previous studies have shown varying results due to different intervention characteristics. This review will examine the efficacy of AOT on clinical cognitive function in stroke.

**Methods**: Randomized controlled trials (RCTs) comparing AOT with non‐AOT interventions in cognitive function were included. Databases searched included PubMed, Cochrane Library, Embase, Web of Science, EBSCO, CNKI, WanFang, and VIP database from inception to May 6, 2024. The risk of bias was assessed using Cochrane's Risk of Bias Assessment Tool 2.0, and the quality of evidence was evaluated with the GRADE approach. RevMan 5.4 and Stata 18.0 were used for the meta‐analysis. After the analysis of cognitive function, meta‐regression was performed to explore the possible sources of heterogeneity. A random‐effects meta‐analysis model using the inverse‐variance and Hartung–Knapp methods was used to calculate pooled estimates and 95% confidence interval (CI) values. We examined the funnel plot and used Egger's regression test to assess for publication bias. This study was conducted by PRISMA reporting guidelines (Appendix S1). The search protocol was prospectively registered in PROSPERO (CRD42024571694).

**Results**: A total of 6 RCTs with 400 participants were included. All the included articles were rated as having B‐level quality. Meta‐analysis showed that AOT significantly improved cognitive function in stroke. Meta‐regression did not find the source of heterogeneity. The GRADE result indicated that the finding was of very low certainty.

**Conclusions**: Cognitive interventions based on AOT can improve cognitive function in stroke patients. However, it should be interpreted cautiously due to heterogeneity and low certainty. To strengthen evidence‐based practices, we advocate for higher‐quality and more homogeneous RCTs, including strict randomization procedures, large sample sizes, extended follow‐up periods, and studies focused on specific disease subtypes.

## Graphical abstract

Action Observation Therapy enhances cognitive function in stroke patients by activating the mirror neuron system. A meta‐analysis of selected studies showed significant improvements in cognitive function, and high‐quality research works in the future are needed.

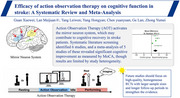

## 1 Introduction

Stroke represents the greatest proportion of the global burden of neurological diseases and ranks second in global mortality (Feigin 2022). In the past 30 years, the individual lifetime risk of stroke has increased by nearly 9% (Prust et al. 2024). While advances in medical technology have steadily increased stroke survival rates, longer survival periods are often accompanied by long‐term sequelae (Feigin et al. 2021). Approximately 1/3 of stroke patients experience poststroke cognitive impairment (PSCI) during the recovery period (Mijajlović et al. 2017). Cognitive function refers to the mental activities that result from the brain receiving and processing external information, transforming it into a process of acquiring or applying knowledge (Harvey 2019). Poststroke cognitive impairment can interfere with functional recovery, the ability to reacquire motor skills, and can significantly reduce independence (Donovan et al. 2008; Nys et al. 2005a; Nys et al. 2005b), ultimately leading to a greatly diminished quality of life and substantial living stress for patients and their families (Zhang and Zhao 2017).

Cognitive impairment may remain or continue to progress and worsen over time, which is why the implementation of interventions to improve cognitive recovery is important (Chuang et al. 2021; Jia et al. 2020; Xu et al. 2024). Poststroke cognitive interventions primarily rely on the mechanisms of neuroplasticity (Carey et al. 2019), enhancing cognitive function through the formation of new synapses (Drigas et al. 2018). The nervous system can adjust and optimize during learning and memory processes via synaptic plasticity, thereby improving the efficiency of information processing and storage (Reis et al. 2009). Consequently, much research on neurorehabilitation techniques aims to promote neuroplasticity to improve cognitive function in stroke patients (Niering and Seifert 2024). Given the importance of neuroplasticity in enhancing cognitive functions and mitigating impairments, researchers are actively exploring various intervention methods.

One such method is Action Observation Therapy (AOT), which is a multisensory approach encompassing motor somatosensory and cognitive rehabilitation (Johansson 2011). When observing others' actions, the neural structures responsible for these actions are activated in the observer's brain as if they were performing the actions themselves (Zhu et al. 2019). For example, the stroke patient is instructed to watch a video showing the action of cleaning: an adult picking up a piece of paper, wiping away dirt from a table, and then throwing the paper into a bin. After observing the video sequence for a time, the individuals may or may not be asked to perform the same action. This activates the mirror neuron system (MNS), which includes the primary motor cortex, the inferior parietal lobule, and the ventral premotor cortex (Rizzolatti 2005; Garrison et al. 2010). Activating the MNS allows the brain to simulate the neural processes involved in executing the observed actions, thereby promoting the formation and strengthening of neural circuits (Urgesi et al. 2006). This can lead to new synaptic connections or the reinforcement of existing ones, directly enhancing neuroplasticity and improving cognitive function (Ryan et al. 2021; Morita et al. 2018). Action observation not only activates the primary motor cortex and ventral premotor cortex but also the prefrontal cortex (Savaki and Raos 2019), which is crucial for cognition (Wojtasik et al. 2020). Therefore, action observation has the potential to enhance cognitive function by boosting the activity of these critical brain regions, indicating the significant potential of AOT in rehabilitation and cognitive function improvement.

Poststroke recovery and rehabilitation require long‐term treatment (Borges et al. 2022), which can place a heavy burden on patients who need to travel back and forth between the hospital and home for interventions. Therefore, it is important to seek affordable and easily applicable therapies for stroke patients. AOT is a noninvasive intervention that can be implemented without location constraints, allowing patients to perform it at home, thus reducing the need for frequent hospital visits.

This review is important as it evaluates the effectiveness of AOT on cognitive function in stroke patients. We will compare the effectiveness of AOT with other conventional cognitive interventions to assess its relative efficacy. By systematically reviewing clinical trials, this study provides evidence to help rehabilitation therapists make informed decisions about using AOT, ultimately improving cognitive outcomes in stroke patients.

## 2 Methods

In this endeavour, a systematic review was meticulously planned and executed, adhering to the guidelines outlined in the Preferred Reporting Items for Systematic Reviews and Meta‐Analyses (PRISMA) statement (Appendix S1). Additionally, this review was prospectively registered in PROSPERO under the registration number CRD42024571694.

### 2.1 Search Methods

A systematic literature search of PubMed, Web of Science, Cochrane Library, Embase, EBSCO, Chinese National Knowledge Infrastructure (CNKI), WANFANG Data Knowledge Service Platform (WanFang), and Weipu Information Chinese Periodical Service Platform (VIP). The search scope was from the establishment of each database to May 6, 2024, including supplementary searches of references and grey literature. The search terms included (cognition OR executive function OR attention OR memory OR perceptual disorders) AND (stroke OR brain ischemia OR brain infarction OR cerebral hemorrhage OR intracranial hemorrhages OR hemiplegia) AND (action observation). The search strategy of each database is in Appendix S2. Literature selection was carried out independently by the two researchers, and any differences were resolved through discussion. If the discussion could not resolve the differences, a third party was consulted for judgment.

### 2.2 Inclusion and Exclusion Criteria

#### 2.2.1 Inclusion Criteria

- Participants

Adults > 18 years diagnosed with stroke, any gender, with any degree of stroke impairment severity, and at any stage of the condition.

- Intervention

AOT (alone or in combination with other therapies).

- Comparisons

Individuals who received non‐AOT intervention, such as usual care, standard care, and placebo intervention.

- Outcomes

The outcome of the study included cognitive assessments such as the Montreal Cognitive Assessment (MOCA) or Mini‐Mental State Examination (MMSE).

- Study Design

Randomized controlled trials (RCTs).

#### 2.2.2 Exclusion Criteria

If sufficient data were not provided, we emailed the corresponding authors to request the information; if we did not receive a response, the study was excluded. We also excluded studies that were descriptive or were conference papers. Additionally, we excluded literature written in languages other than Chinese and English.

### 2.3 Data Extraction

The eligible studies were screened by two investigators according to the inclusion criteria, and the detailed information was extracted and cross‐checked. If there was any disagreement, the two researchers discussed and asked for the assistance of a third investigator. Detailed information collected for each paper included: study information (author's name, publication year, and publication country), participant's characteristics (sample size, gender, type of stroke, duration of illness, years of education, and age), intervention characteristics, and cognitive outcomes.

### 2.4 Risk of Bias

The Cochrane's Risk of Bias Assessment Tool 2.0 (ROB2) was used to assess the risk of bias in the included studies by two investigators independently, focusing on sequence generation; allocation concealment; blinding of participants, personnel and outcome assessors; incomplete outcome data; selective outcome reporting; and other sources of bias (Cumpston et al. 2019). Each item of risk bias was divided into “low risk of bias,” “high risk of bias,” or “unclear risk of bias.” If the two researchers could not reach an agreement, a third impartial researcher was engaged to provide a definitive judgment.

### 2.5 Quality of Evidence

Two investigators also assessed the quality of evidence by using the “Grading of Recommendations Assessment, Development and Evaluation” (GRADE) (Guyatt et al. 2011). Evidence was assessed and classified as “high,” “moderate,” “low,” and “very low.” The assessment included risk of bias, inconsistency, indirectness, imprecision and other considerations. Any disagreement was resolved through discussion and consultation with a third reviewer.

### 2.6 Statistical Analysis

Meta‐analysis was performed using RevMan 5.4 and Stata 18.0 software, and the results were displayed using forest plots. The outcome measures in this study are continuous variables; therefore, the mean difference (MD) or standardized mean difference (SMD) was used as the effect size, with 95% confidence intervals (CI) obtained from forest plots for analysis. Since DerSimonian and Laird is the default random‐effects model in RevMan 5.4. This effects model was employed with the estimates of heterogeneity taken from the inverse‐variance random‐effects model. Results from the DerSimonian and Laird method were then converted to results using the Hartung‐Knapp‐Sidak‐Jonkman method via the procedures outlined in (Inthout et al. 2014). This post hoc conversion was employed because the Hartung–Knapp–Sidak–Jonkman method yields more adequate error rates for random effects meta‐analyses using a relatively small number of studies (Inthout et al. 2014). The conversion of results between methods was carried out using Microsoft Excel v.15.26 (Redmond, WA, USA). The *I* ^2^ statistic was used to assess heterogeneity among the included studies. The Cochrane Handbook provides a rough yet widely used rule to interpret this measure: *I* ^2 ^≤  40% may indicate unimportant heterogeneity, 30%  ≤ *I* ^2 ^≤  60% may represent moderate heterogeneity, 50%  ≤ *I* ^2 ^≤ 90% may represent substantial heterogeneity, and 75%  ≤*I* ^2 ^≤  100% implies considerable heterogeneity (Deeks et al. 2019). To explore the sources of heterogeneity and gain a better insight into the effects of the interventions, subgroup analysis, and meta‐regression were used. Sensitivity analysis was performed by sequentially excluding individual studies to assess the robustness of the synthesized results. Funnel plots and Egger bias tests were used to assess publication bias.

## 3 Results

### 3.1 Search Results

**FIGURE 1:**
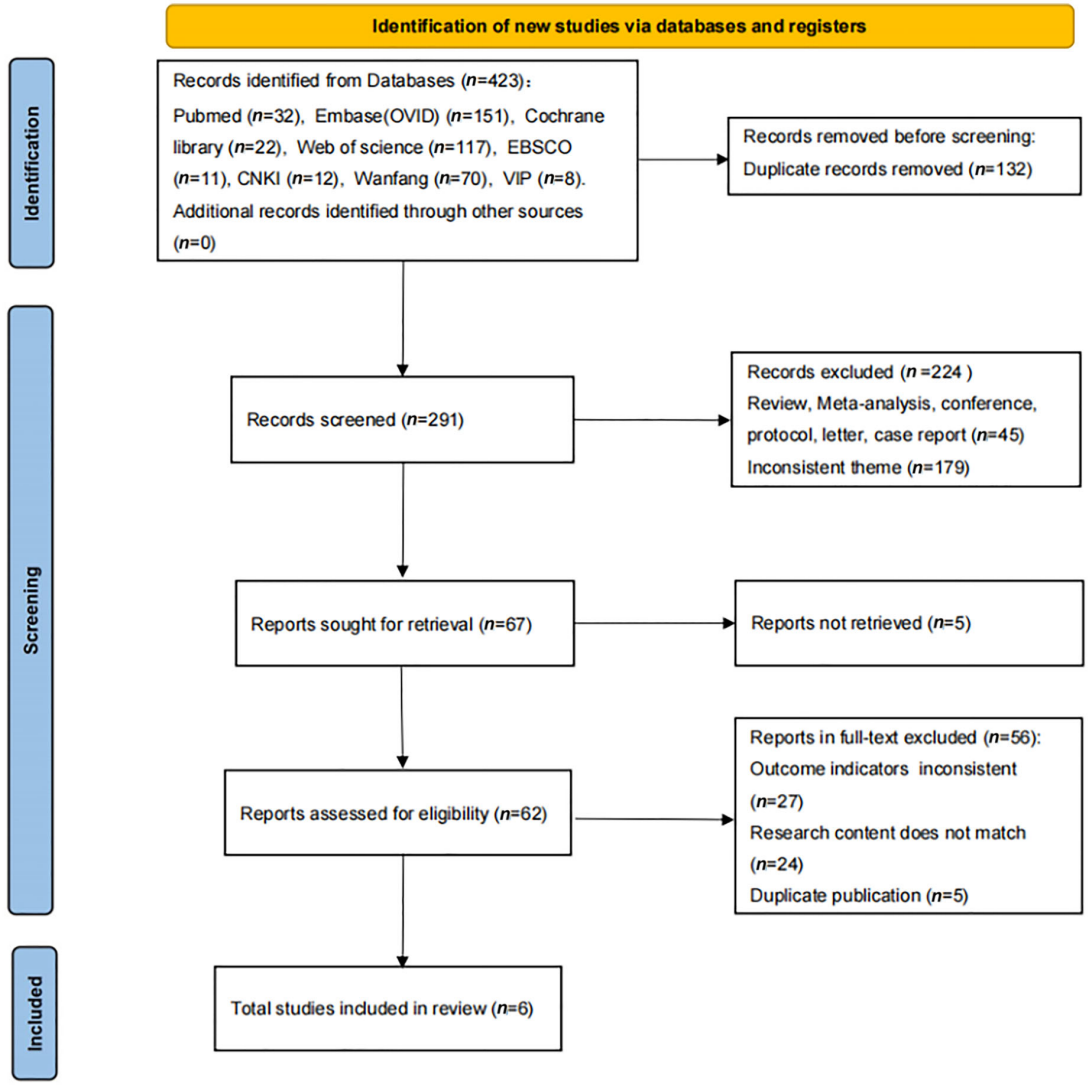
PRISMA flow diagram of literature screening and selection process.

The flowchart of the study selection process is presented in Figure 1. We searched 8 databases and found 423 articles. After removing duplicates, 291 articles remained. After reading titles and abstracts, 224 articles were excluded. With 5 articles not retrieved, 62 remained for full‐text review. Upon reviewing the full texts, 56 studies were excluded based on our inclusion and exclusion criteria. Ultimately, six full‐text studies were included in the qualitative synthesis.

### 3.2 Study Characteristics

The characteristics of the included studies were presented in Table 1. The final sample was composed of six RCTs. The sample size for each study ranged from 30 to 90 participants, with a total of 400 participants across all studies. The mean ages of the participants ranged from 46.47 to 65.2 years. Totally 3 studies examined hemorrhagic stroke and ischemic stroke as the study population; while three studies did not report the type of stroke. Each session lasted 20–30 min, with total intervention duration ranging from 3 to 8 weeks. We extracted all group data from each study but ultimately included only the two groups that aligned with the PICO (Population, Intervention, Comparison, and Outcome) criteria for this meta‐analysis. This approach ensured that the data included were directly relevant to the research question, enhancing the accuracy and interpretability of the results.

**TABLE 1:** Characteristics of the included studies.

Basic features of the included literature														
**Author, year**	**Country**	**Gender** **Male:Female**	**Research subjects**	**Control group / intervention group (cases)**	**Average age (years, control group / experimental group)**	**The type of stroke**	**Duration of illness**	**Years of education**	**Control group**	**Intervention group**	**Dosage** **(time)**	**Frequency**	**Duration time**	**Outcome**
(Liu et al. 2022)	China	C:6:4 E1:8:2 E2:5:5	VCI after stroke(MMSE ≤ 26)	10/10/10	C:(64.60 ± 9.81) E1:(64.90 ± 9.15) E2:(65.20 ± 10.12)	Not reported	C:7.00 ± 3.27(w) E1:6.90 ± 2.33(w) E2:7.40 ± 2.72(w)	C:10.00 ± 2.94 E1:9.40 ± 2.72 E2:9.90 ± 2.77	C: conventional cognitive treatment	E1: MI + AOT E2: Conventional cognitive treatment + MI + AOT	20 min	1 time/day, 5 days/1 week	8 weeks	MOCA
(Mao et al. 2020)	China	C:15:15 E:16:14	Stroke(MoCA ≥ 15)	30/30	C:(57 ± 6) E:(54 ± 7)	1.Hemorrhagic stroke(C:6; E:7) 2.Ischemic stroke(C:24; E:23)	C:6 ± 2(m) E:6 ± 2(m)	C:12 ± 3 E:12 ± 3	C: routine upper limb rehabilitation training and Schulte Grid training	E: Same upper limb rehabilitation training + AOT	20 min	5 times/1 week	8 weeks	MOCA
(Shen et al. 2018)	China	C:13:9 E:13:9	Stroke(MMSE > 17)	22/22	C:(52.04 ± 12.86) E:(54.09 ± 10.41)	1.Hemorrhagic stroke(C:10; E:14) 2.Ischemic stroke(C:12; E:8)	C:36.09 ± 19.73(d) E:34.95 ± 15.93(d)	Not reported	C: routine limb training and occupational treatment + irregular video watching	E: Routine limb training and occupational treatment + AOT	30 min	5 times/1 week	3 weeks	MMSE
Wang (2022)	China	C:22:21 E:19:24	Stroke(MMSE ≤ 26)	43/43	C:(60.25 ± 2.36) E:(60.35 ± 2.14)	Not reported	C:1.34 ± 0.19(m) E:1.56 ± 0.21(m)	Not reported	C: conventional nutritional support and conventional drug therapy + rTMS	E: Conventional nutritional support and conventional drug therapy + rTMS + AOT	30 min	2 times/day, 5 days/1 week	4 weeks	MOCA MMSE
(Wu et al. 2022)	China	C:14:16 E1:12:18 E2:13:17	Stroke(MMSE ≤ 26)	30/30/30	C:(56.80 ± 11.70) E1:(54.20 ± 12.80) E2:(55.40 ± 11.10)	1.Hemorrhagic stroke(C:18; E1:20; E2:21) 2.Ischemic stroke(C:12; E1:10; E2:9)	C:88.70 ± 29.20(d) E1:86.30 ± 29.70(d) E2:85.60 ± 31.10(d)	C:11.80 ± 3.00 E1:11.20 ± 3.40 E2:12.00 ± 3.80	C: routine limb training and conventional cognitive treatment	E1: Routine limb training and conventional cognitive treatment + rTMS E2: Routine limb training and conventional cognitive treatment + rTMS + AOT	30 min	2 times/day, 5 days/1 week	4 weeks	MMSE MOCA
(Yang 2020)	China	C:12:18 E1:12:18 E2:4:26	Stroke(MMSE < 24)	30/30/30	C:(50.80 ± 11.02) E1:(46.67 ± 11.94) E2:(52.40 ± 12.72)	Not reported	C:73.40 ± 72.94(d) E1:107.87 ± 103.35(d) E2:109.73 ± 103.23(d)	C:11.87 ± 2.90 E1:12:33 ± 3.44 E2:12.07 ± 2.79	C: conventional cognitive treatment	E1: conventional cognitive treatment + AOT E2: conventional cognitive treatment+ AOT + rTMS	30 min	1 time/day, 5 days/1 week	4 weeks	MOCA MMSE

Abbreviations: C, control; d, day(s); E, experimental; m, month(s); MI, motor imagery; MMSE, Mini‐Mental State Examination; MoCA, Montreal Cognitive Assessment Scale; rTMS, repetitive transcranial magnetic stimulation; VCI, vascular cognitive impairment; w, week(s).

### 3.3 Study Quality

**FIGURE 2:**
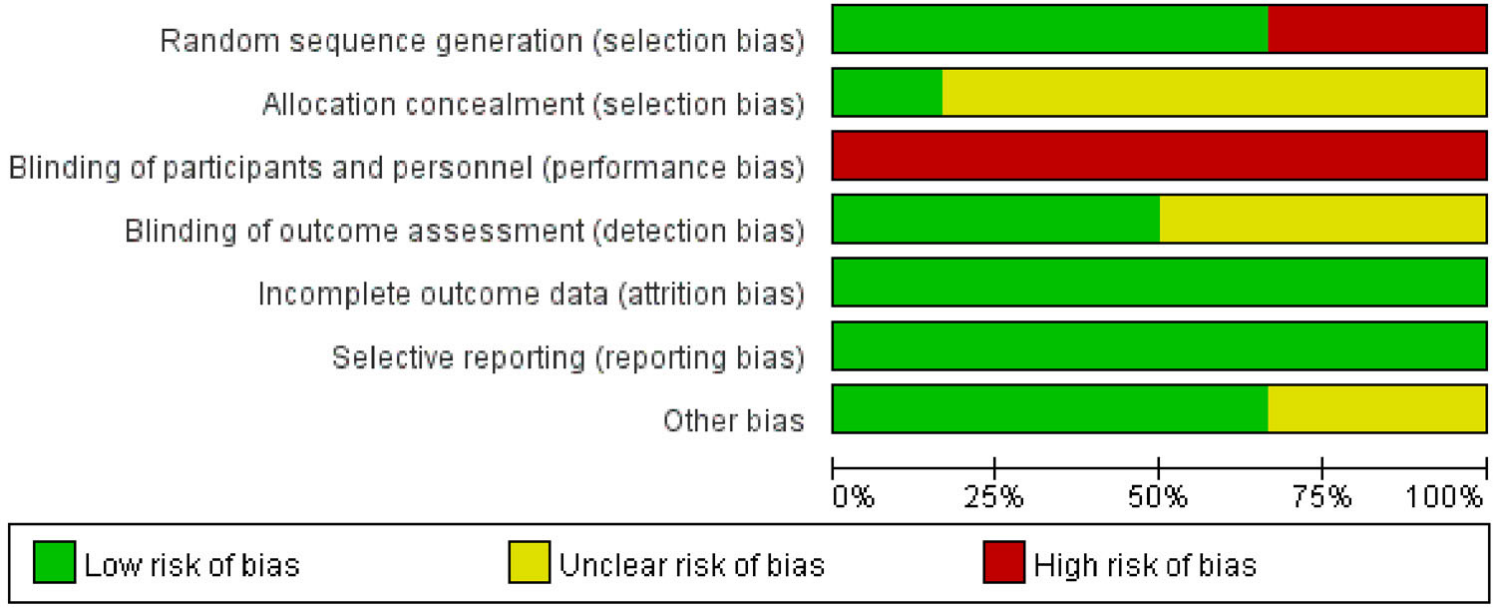
The Cochrane risk‐of‐bias assessment for included studies.

**FIGURE 3:**
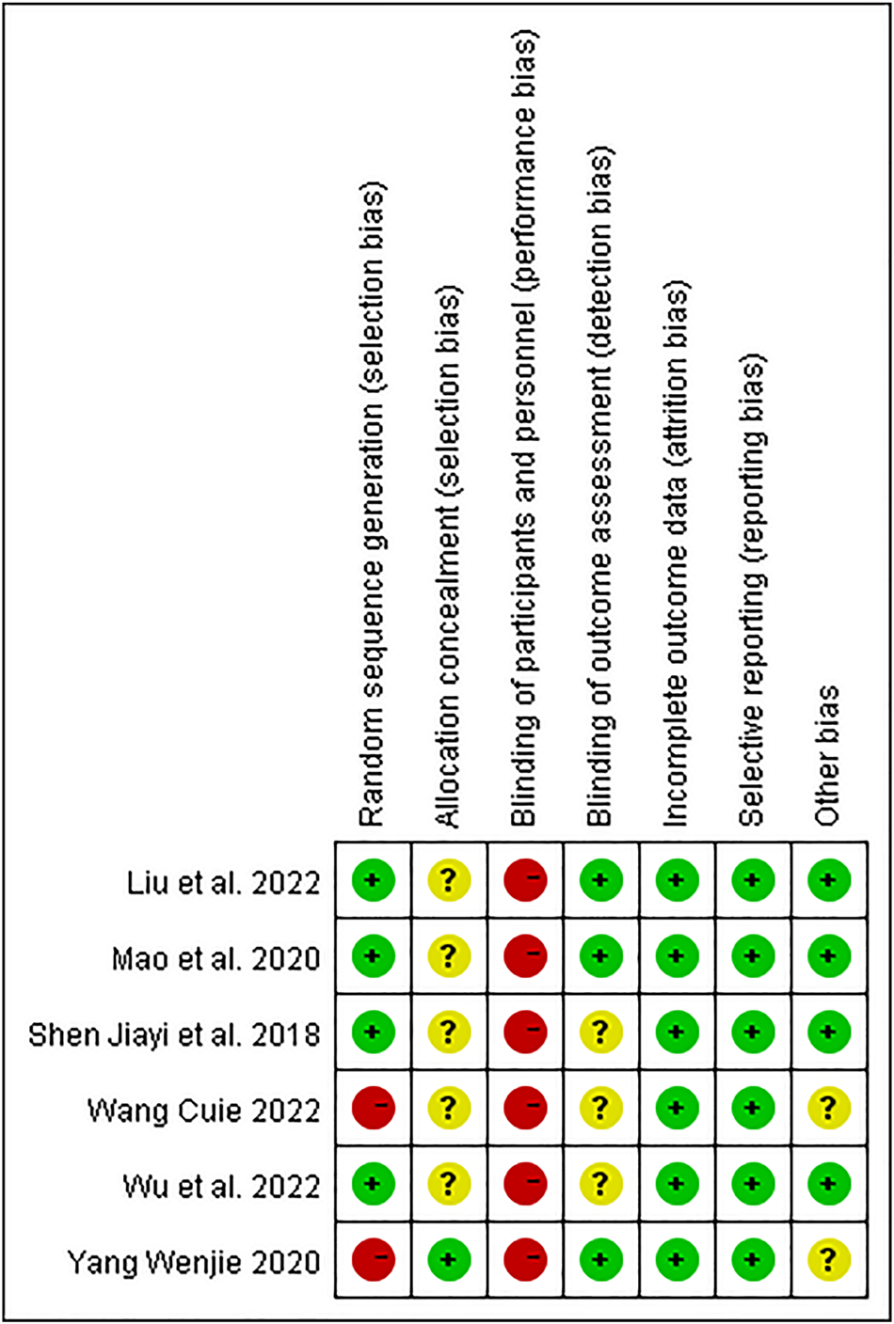
Summary of the Cochrane risk‐of‐bias judgments across all included studies.

The Cochrane risk of bias of the included studies is shown in Figures 2 and Figure 3.

Four (Liu et al. 2022; Mao et al. 2020; Shen et al. 2018; Wu et al. 2022) studies reported methods of generating random sequences to be judged as having a low risk of bias, two (Shen et al. 2018; Wu et al. 2022) of which referred to a random number and two (Liu et al. 2022; Mao et al. 2020) of which used a computer random number generator. Because one (Wang 2022) study was grouped by type of treatment and one (Yang 2022) by time of admission, a total of two (Wang 2022; Yang 2022) articles were judged to be at high risk of selection bias. One (Mao et al. 2020) mentioned allocation concealment, and others did not. Because the intervention in this study could not be blinded to participants, all RCTs (Liu et al. 2022; Mao et al. 2020; Shen et al. 2018; Wang 2022; Wu et al. 2022; Yang 2022) had a high risk of performance bias. Three (Liu et al. 2022; Mao et al. 2020; Yang 2022) studies mentioned blinding of outcome assessment, and the rest did not. One (Liu et al. 2022) study reported reasons for withdrawal and loss of follow‐up, while the remaining studies had no patient dropouts or loss to follow‐up, so all the attrition bias was judged as low risk. All the studies reported whether baseline characteristics were comparable between the experimental group and the control group. Four (Liu et al. 2022; Mao et al. 2020; Shen et al. 2018; Wu et al. 2022) studies mentioned there were no interest conflicts, and others were not mentioned, leading to uncertainty about other potential sources of bias in these two studies.

### 3.4 Efficacy Analysis

#### 3.4.1 Cognitive Function

Two (Liu et al. 2022; Mao et al. 2020) studies used only MoCA, one (Shen et al. 2018) study used only MMSE and three (Wang 2022; Wu et al. 2022; Yang 2022) studies used both MoCA and MMSE. As for the multi‐arm studies, we only extracted two groups of data that could be used for meta‐analysis. Since MoCA is more sensitive and more applicable than MMSE in the cognitive investigation of stroke (Salvadori et al. 2013; Godefroy et al. 2011; Burton and Tyson 2015), we only conducted a meta‐analysis of the five RCTs using MoCA.

**FIGURE 4:**
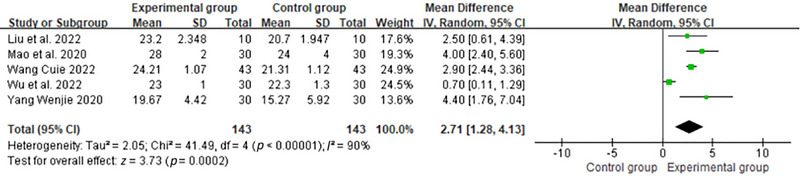
Forest plot of meta‐analysis for cognitive function outcomes.

The random effect model of meta‐analysis of five articles using the MoCA scale showed that the experimental group was superior to the control group in improving individuals' cognitive function, and the difference was statistically significant (MD = 2.71, 95% CI: 1.28 to 4.13, *I* ^2^ = 90%, *p* < 0.01) (Figure 4). The results from the DerSimonian and Laird method were converted to results using the Hartung‐Knapp‐Sidak‐Jonkman method, which slightly altered the precision of the MD estimate (95% CI: 0.89 to 4.53). Using the Hartung‐Knapp‐Sidak‐Jonkman method, the model *t*‐statistic = 4.14 (*p* < 0.01) (Appendix S3).

The certainty of evidence for AOT to improve cognitive function in stroke was “low” (Table 2).

**TABLE 2:** GRADE evidence profile for cognitive function outcome.

**Certainty assessment**							**No. of patients**		**Effect**		**Certainty**
**No of studies**	**Study design**	**Risk of bias**	**Inconsistency**	**Indirectness**	**Imprecision**	**Other considerations**	**Paro**	**Control group**	**Relative (95% CI)**	**Absolute (95% CI)**	
**Cognitive function**											
5	Randomized trials	Serious^a^	Not serious	Not serious	Serious^b^	None	143	143	—	MD **2.71 higher** (1.28 higher to 4.13 higher)	⨁⨁◯◯ Low

^a^Downgrade one level for the risk of bias because of the result of the Cochrane's Risk of Bias.

^b^Downgrade one level for the risk of bias because of the simple size less than 400.

#### 3.4.2 Meta‐Regression

In order to explore the source of heterogeneity, the meta‐regression analysis was conducted to examine whether the intervention characteristics, minutes per session, number of weeks, overall minutes of therapy, and frequency were associated with the observed heterogeneity in the cognitive function. The result indicated that all of them did not significantly affect heterogeneity (*p* > 0.05; Table 3).

**TABLE 3:** Meta‐regression analysis of potential sources of heterogeneity for cognitive function outcome.

Characteristics	Coefficient	*t*	*p*	95%CI
Intervention	−1.76	−1.43	0.25	[−5.68, 2.16]
Minutes per session	−0.93	−0.63	0.57	[−5.62, 3.77]
Number of weeks	0.93	0.63	0.57	[−3.77, 5.62]
Overall minutes of therapy	−1.35	−1.57	0.21	[−4.09, 1.38]
Frequency	−1.76	−1.43	0.25	[−5.68, 2.16]

### 3.5 Publication Bias and Sensitive Estimate

**FIGURE 5:**
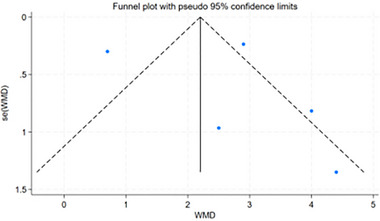
Funnel plot assessing publication bias for the meta‐analysis.

**FIGURE 6:**
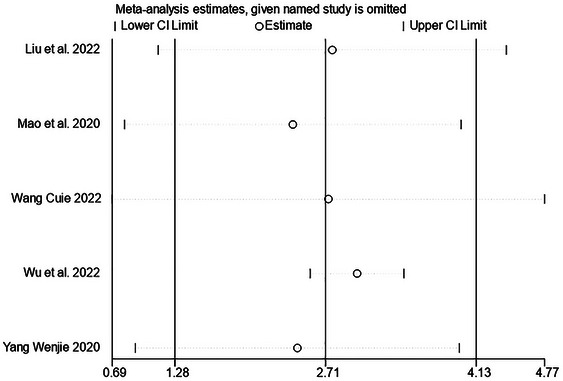
Sensitivity analysis evaluating the robustness of meta‐analysis results.

**FIGURE 7:**
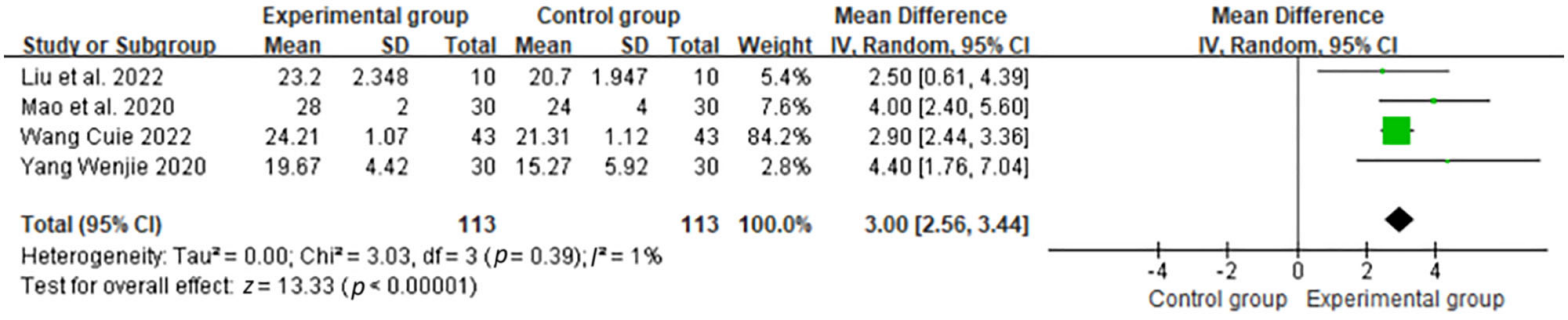
Forest plot of sensitivity analysis after exclusion of the study by Wu et al. (2022).

The funnel plot is displayed in Figure 5. The Egger test (*t* = 0.47, *p =* 0.67) did not indicate publication bias. In terms of the robustness analysis of the review, the study used a case‐by‐case elimination approach to demonstrate that when the study by (Wu et al. 2022) was removed, there was low statistical heterogeneity (*p* = 0.39, *I* ^2^ = 1%). And the AOT group still had a significant difference in improving cognitive function (MD = 3.00, 95% CI: 2.56 to 3.44, *p* < 0.01), which indicated that the RCT (Wu et al. 2022) was the source of the heterogeneity (Figures 6 and 7).

## 4 Discussion

The current literature shows that AOT can improve cognitive function in stroke, but the effects are mixed. This is the first systematic review and meta‐analysis to evaluate the effectiveness of AOT for improving cognitive function in stroke patients. This study includes six RCTs with 400 patients. We found that AOT can improve cognitive function in stroke patients. Furthermore, the results of sensitivity analyses confirmed the robustness of the results. However, because the results of the meta‐analysis were heterogeneous, we conducted a further meta‐regression to try to find the source of heterogeneity, but the result was not satisfactory. The quality of evidence was low, so the conclusion should be treated with caution.

Action observation is inherently a cognitive process where individuals observe others' behaviors to understand, select, and imitate the forms and actions (Buchignani et al. 2019). When observing others, the MNS is activated, creating a “resonance” in the motor system (Rizzolatti and Craighero 2004). This resonance facilitates understanding the intentions behind actions, aids in task execution, and clarifies the relationship between specific actions and outcomes, thereby promoting the learning and acquisition of motor skills (Urgesi et al. 2006; Petrosini et al. 2003). The brain areas activated during action observation form the action observation network, which includes the ventral and dorsal premotor cortex, superior parietal lobule, inferior parietal lobule, superior temporal sulcus, and dorsolateral prefrontal cortex (Mizuguchi and Kanosue 2017). A subset of these regions, namely the ventral premotor cortex, inferior parietal lobule, and superior temporal sulcus, constitutes the core of the mirror neuron system. The superior temporal gyrus encodes and decodes action‐related information (Vander Wyk et al. 2009), while the dorsolateral prefrontal cortex plays a crucial role in working memory during action planning, helping to maintain and process memory content and ensuring that behavioral goals remain unaffected by external distractions (Blumenfeld and Ranganath 2006). Additionally, the posterior parietal cortex is involved in encoding, enhancing attention, and receiving task‐relevant information (Shomstein 2012). This distributed and hierarchical circuit neural circuit is essential for cognitive improvement (Courtney et al. 1998).

Although the meta‐analysis result showed the positive impact of AOT on cognitive function, the result exhibited high heterogeneity, and we failed to identify the source of it. This may be due to the limited number of studies included (only five articles reporting MoCA scores) in the meta‐regression, and the sample size for the meta‐regression was relatively small, comprising only 286 participants. In small sample studies, random error can cause fluctuations in the estimates, making it difficult for regression analysis to distinguish between true effects and random errors (Ito et al. 1986). Furthermore, although all included studies focused on stroke patients, the basic characteristics of the populations differed (such as stroke type, disease stage, etc.). Due to the limited number of studies, the model may not have been able to account for all sources of variability, which contributed to the inability to effectively explain the heterogeneity (Schmid et al. 2004). Additionally, with small sample sizes, low statistical power reduces the ability of the regression analysis to detect small effects. Even when significant sources of heterogeneity exist, the analysis might fail to detect these relationships due to the insufficient sample size, leading to a higher likelihood of Type II errors (false negatives) (Hedges and Pigott 2004).

Therefore, to obtain more comprehensive and reliable evidence, larger sample sizes are needed in future studies to determine the effectiveness of the intervention. It is important to note that future research should clearly define the procedures for randomization, allocation concealment, and blinding and strictly adhere to the requirements of RCTs. To enhance the robustness of future studies, we advocate for higher‐quality and more homogeneous RCTs, including strict randomization processes, large sample sizes, and extended follow‐up periods. The use of imaging tools such as electroencephalographic and magnetic resonance imaging could also be incorporated to provide more objective data. Additionally, subtype studies can be conducted, such as those based on stroke type (ischemic or hemorrhagic) and disease stage (acute or chronic), to obtain higher quality information and thus strengthen the evidence‐based practices in this field.

Although there is a lack of high‐quality evidence‐based support for the impact of AOT on cognitive function in stroke patients. Considering the neuromechanism by which AOT promotes motor relearning through the activation of the mirror neuron system (Simone et al. 2017), AOT may be a highly valuable and promising cognitive intervention for stroke patients. Due to the inclusion of studies with small sample sizes, the precision and robustness of the pooled effect size may have been affected, thus limiting the validity of the finding. However, the result of this study can be considered as providing a foundation for future investigations into effective PSCI.

## 5 Limitation

Our study had several limitations. First, we did not report the agreement measures of the selection process, risk of bias assessment, or the GRADE evaluation. Second, the quality of evidence was low because the small sample sizes of the included studies might have led to over or underestimation of the treatment effect. Finally, due to insufficient data, the meta‐analysis comparisons included the outcomes at the end time points of the AOT only, so we cannot find the long‐term effects of AOT on cognitive function.

## 6 Conclusion

This systematic review found that AOT is effective in improving cognitive function in stroke patients, however, the evidence was considered low according to GRADE, so conclusions should be treated with caution. Future research needs to optimize study designs and conduct more high‐quality, large‐sample, multicenter, RCTs to provide robust evidence for the effectiveness of AOT in cognitive interventions for stroke patients.

## Author Contributions

**Xuewei Guan**: conceptualization, methodology, software, data curation; writing – original draft. **Meijuan Lan**: conceptualization, supervision, writing – review and editing. **Leiwen Tang**: conceptualization, methodology, writing – review and editing. **Hongyan Yang**: writing – review and editing. **Yuanyuan Chen**: software, funding acquisition. **Lan Ge**: data curation. **Yumei Zhong**: data curation.

### Peer Review

The peer review history for this article is available at https://publons.com/publon/10.1002/brb3.70474

## Supporting information



Supporting Information

Supporting Information

Supporting Information

## Data Availability

The data supporting the findings of this study are available from the corresponding author upon request.
