# Supplementary material for: Efficacy of Action Observation Therapy on Cognitive Function in Stroke: A Systematic Review and Meta‐Analysis
Source: Brain Behav. 2025 Apr 21;15(4):e70474. doi: 10.1002/brb3.70474 (PMC12012254; doi:10.1002/brb3.70474)
Supplement: Supplementary file 2 — Supporting Information [file BRB3-15-e70474-s002.docx]

| Pubmed | | Total |
| --- | --- | --- |
| #1 | "action observation"[Title/Abstract] OR "action observation training"[Title/Abstract] OR "action observation treatment"[Title/Abstract] OR "action observation therapy"[Title/Abstract] OR "action observation-execution"[Title/Abstract] OR "motor observation"[Title/Abstract] OR "movement observation"[Title/Abstract] OR "motion observation"[Title/Abstract] OR "gesture observation"[Title/Abstract] | 1953 |
| #2 | ("Perceptual Disorders"[Mesh]) OR ("visual perception"[Title/Abstract] OR "visual construct"[Title/Abstract] OR agnosia[Title/Abstract] OR prosopagnosia[Title/Abstract] OR stereognosis[Title/Abstract] OR "auditory perception"[Title/Abstract] OR "perceptual distortion"[Title/Abstract] OR "perceptual motor processes"[Title/Abstract] OR "time perception"[Title/Abstract] OR "visual perception"[Title/Abstract] OR "perceptual disturbances"[Title/Abstract] OR "sensory integration dysfunction"[Title/Abstract] OR hemineglect[Title/Abstract] OR "hemi-neglect"[Title/Abstract] OR "unilateral neglect"[Title/Abstract] OR "spatial neglect"[Title/Abstract] OR "spatial-neglect"[Title/Abstract] OR "hemi-attention"[Title/Abstract] OR "hemi attention"[Title/Abstract] OR visuospatial[Title/Abstract] OR "receptive fields"[Title/Abstract] OR "sensory neglect"[Title/Abstract]) | 69333 |
| #3 | ("Memory"[Mesh]) OR (forgetting[Title/Abstract] OR "episodic memory"[Title/Abstract] OR "explicit memory"[Title/Abstract] OR "implicit memory"[Title/Abstract] OR "long term memory"[Title/Abstract] OR "short term memory"[Title/Abstract] OR "cognitive aging"[Title/Abstract] OR "memory training"[Title/Abstract]) | 180873 |
| #4 | ("Attention"[Mesh]) OR (concentration[Title/Abstract] OR vigilance[Title/Abstract] OR inattention[Title/Abstract] OR distract*[Title/Abstract] OR awareness[Title/Abstract] OR "divided attention"[Title/Abstract] OR "focused attention"[Title/Abstract] OR "selective attention"[Title/Abstract] OR "sustained attention"[Title/Abstract] OR "visual attention"[Title/Abstract] OR "attention span"[Title/Abstract]) | 1786092 |
| #5 | ("Executive Function"[Mesh]) OR ("executive dysfunction"[Title/Abstract] OR "dysexecutive syndrome"[Title/Abstract] OR "dysexecutive function"[Title/Abstract] OR "concept formation"[Title/Abstract] OR "goal management"[Title/Abstract] OR "cognitive flexibility"[Title/Abstract] OR "inhibition control"[Title/Abstract] OR "working memory"[Title/Abstract]) | 64799 |
| #6 | ("Cognition"[Mesh]) OR ("cognitive disorder"[Title/Abstract] OR "cognitive disruption"[Title/Abstract] OR "cognitive impair*"[Title/Abstract] OR confusion[Title/Abstract] OR "neurobehavioral manifestation"[Title/Abstract] OR "neurobehavioral disorder"[Title/Abstract] OR "cognitive ability"[Title/Abstract] OR "neurobehavioral disruption"[Title/Abstract]) | 337108 |
| #7 | #2 OR #3 OR #4 OR #5 OR #6 | 2268182 |
| #8 | ("Stroke"[Mesh]) OR (poststroke[Title/Abstract] OR post-stroke[Title/Abstract] OR "cerebrovascular disorder"[Title/Abstract] OR cerebrovascular[Title/Abstract] OR "cerebral vascular"[Title/Abstract] OR "cerebrovascular disease"[Title/Abstract] OR "basal ganglia cerebral vascular disease"[Title/Abstract] OR CVA[Title/Abstract] OR "cerebrovascular accident"[Title/Abstract]) | 256934 |
| #9 | ("Brain Ischemia"[Mesh]) OR ("ischemic encephalopathy"[Title/Abstract] OR "cerebral ischemia"[Title/Abstract] OR "carotid artery disease"[Title/Abstract] OR "intracranial arterial disease"[Title/Abstract]) | 141790 |
| #10 | ("Brain Infarction"[Mesh]) OR ("brain infarct*"[Title/Abstract] OR "cerebral infarct*"[Title/Abstract] OR "cerebral thrombo*"[Title/Abstract] OR "cerebral emboli*"[Title/Abstract]) | 64444 |
| #11 | ("Cerebral Hemorrhage"[Mesh]) OR ("Cerebral haemorrhag*"[Title/Abstract] OR "brain bleed*"[Title/Abstract]) | 39547 |
| #12 | ( "Intracranial Hemorrhages"[Mesh]) OR ("intracranial haemorrhag*"[Title/Abstract] OR "intracranial hemorrhag*"[Title/Abstract] OR "subarachnoid haemorrhag*"[Title/Abstract] OR "subarachnoid hemorrhag*"[Title/Abstract] OR "intracerebral haemorrhag*"[Title/Abstract] OR "intracerebral hemorrhag*"[Title/Abstract] OR "subdural haemorrhage*"[Title/Abstract] OR "subdural hemorrhag*"[Title/Abstract] OR "extradural haemorrhage*"[Title/Abstract] OR "extradural hemorrhag*"[Title/Abstract]) | 109524 |
| #13 | ("Hemiplegia"[Mesh]) OR (hemiplegia*[Title/Abstract] OR hemiparesis*[Title/Abstract] OR paresis*[Title/Abstract]) | 39226 |
| #14 | #8 OR #9 OR #10 OR #11 OR #12 OR #13 | 438890 |
| #15 | #1 AND #7 AND 14 | 32 |

| Cochrane Library | | Total |
| --- | --- | --- |
| #1 | ("Action observation" OR "Action observation training" OR "Action observation treatment" OR "Action observation therapy" OR "Action observation-execution" OR "Motor observation" OR "Movement observation" OR "Motion observation" OR "Gesture observation"):ti,ab,kw | 543 |
| #2 | MeSH descriptor: [Stroke] explode all trees | 17473 |
| #3 | (poststroke OR post-stroke OR "cerebrovascular disorder" OR "cerebrovascular accident" OR "cerebral vascular" OR "basal ganglia cerebral vascular disease" OR CVA OR "cerebrovascular accident"):ti,ab,kw | 33872 |
| #4 | MeSH descriptor: [Brain Ischemia] explode all trees | 5899 |
| #5 | ("ischemic encephalopathy" OR "cerebral ischemia" OR "carotid artery disease" OR "Intracranial arterial disease"):ti,ab,kw | 2423 |
| #6 | MeSH descriptor: [Brain Infarction] explode all trees | 5899 |
| #7 | ("Brain infarct" OR "cerebral infarct" OR "cerebral thrombus" OR "cerebral embolism"):ti,ab,kw | 368 |
| #8 | MeSH descriptor: [Cerebral Hemorrhage] explode all trees | 1580 |
| #9 | ("cerebral hemorrhage" OR "brain bleed"):ti,ab,kw | 2825 |
| #10 | MeSH descriptor: [Intracranial Hemorrhages] explode all trees | 3162 |
| #11 | ("intracranial hemorrhage" OR "subarachnoid hemorrhage" OR "intracerebral hemorrhage" OR "subdural hemorrhage" OR "extradural hemorrhage"):ti,ab,kw | 7165 |
| #12 | MeSH descriptor: [Hemiplegia] explode all trees | 982 |
| #13 | ("hemiplegia" OR "hemiparesis" OR "paresis"):ti,ab,kw | 6560 |
| #14 | (#2 OR #3) | 44124 |
| #15 | (#4 OR #5) | 7610 |
| #16 | (#6 OR #7) | 2196 |
| #17 | (#8 OR #9) | 2873 |
| #18 | (#10 OR #11) | 8254 |
| #19 | (#12 OR #13) | 6560 |
| #20 | (#14 OR #15 OR #16 OR #17 OR #18 OR #19) | 56346 |
| #21 | MeSH descriptor: [Cognition] explode all trees | 16241 |
| #22 | ("cognitive disorder" OR "cognitive disruption" OR "cognitive impairment" OR confusion OR "neurobehavioral manifestation" OR "neurobehavioral disorder" OR "cognitive ability" OR "neurobehavioral disruption"):ti,ab,kw | 17616 |
| #23 | MeSH descriptor: [Executive Function] explode all trees | 1841 |
| #24 | ("executive dysfunction" OR "dysexecutive syndrome" OR "dysexecutive function" OR "concept formation" OR "goal management" OR "cognitive flexibility" OR "inhibition control" OR "working memory"):ti,ab,kw | 8613 |
| #25 | (Attention):ti,ab,kw | 41343 |
| #26 | (concentration OR vigilance OR inattention OR distraction OR awareness OR "divided attention" OR "focused attention" OR "selective attention" OR "sustained attention" OR "visual attention" OR "attention span"):ti,ab,kw | 138129 |
| #27 | MeSH descriptor: [Memory] explode all trees | 9947 |
| #28 | (forgetting OR "episodic memory" OR "explicit memory" OR "implicit memory" OR "long term memory" OR "short term memory" OR "cognitive aging" OR "memory training"):ti,ab,kw | 4957 |
| #29 | MeSH descriptor: [Perceptual Disorders] explode all trees | 1404 |
| #30 | ("visual perception" OR "visual construct" OR agnosia OR prosopagnosia OR stereognosis OR "auditory perception" OR "perceptual distortion" OR "perceptual motor processes" OR "time perception" OR "visual perception" OR "perceptual disturbances" OR "sensory integration dysfunction" OR "hemi-neglect" OR "unilateral neglect" OR "spatial neglect" OR "hemi-attention" OR "visuospatial" OR "receptive fields" OR "sensory neglect"):ti,ab,kw | 6416 |
| #31 | (#21 OR #22) | 32236 |
| #32 | (#23 OR #24) | 9819 |
| #33 | (#25 OR #26) | 171089 |
| #34 | (#27 OR #28) | 13066 |
| #35 | (#29 OR #30) | 7318 |
| #36 | (#31 OR #32 OR #33 OR #34 OR #35) | 208801 |
| #37 | (#1 AND #20 AND #36) | 22 |

| Embase | | Total |
| --- | --- | --- |
| #1 | ‘action observation therapy’/exp | 29 |
| #2 | ‘action observation’:ti,ab,kw OR ‘action observation training’:ti,ab,kw OR ‘action observation treatment’:ti,ab,kw OR ‘action observation therapy’:ti,ab,kw OR ‘action observation execution’:ti,ab,kw OR ‘motor observation’:ti,ab,kw OR ‘movement observation’:ti,ab,kw OR ‘motion observation’:ti,ab,kw OR ‘gesture observation’:ti,ab,kw | 2365 |
| #3 | #1 OR #2 | 2365 |
| #4 | ‘cerebrovascular accident’/exp | 453841 |
| #5 | poststroke:ti,ab,kw OR ‘post stroke’:ti,ab,kw OR ‘cerebrovascular disorder’:ti,ab,kw OR cerebrovascular:ti,ab,kw OR ‘cerebrovascular disease’:ti,ab,kw OR ‘basal ganglia cerebral vascular disease’:ti,ab,kw OR ‘cerebrovascular accident’:ti,ab,kw | 158649 |
| #6 | #4 OR #5 | 545446 |
| #7 | ‘brain ischemia’/exp | 222853 |
| #8 | ‘ischemic encephalopathy’:ti,ab,kw OR ‘cerebral ischemia’:ti,ab,kw OR ‘carotid artery disease’:ti,ab,kw OR ‘intracranial arterial disease’:ti,ab,kw | 70959 |
| #9 | #7 OR #8 | 244650 |
| #10 | ‘brain infarction’/exp | 94268 |
| #11 | ‘brain infarct*’:ti,ab,kw OR ‘cerebral infarct’:ti,ab,kw OR ‘cerebral thromb*’:ti,ab,kw OR ‘cerebral emboli*’:ti,ab,kw | 48027 |
| #12 | #10 OR #11 | 110402 |
| #13 | "brain hemorrhage'/exp | 192478 |
| #14 | ‘cerebral haemorrhag*’:ti,ab,kw OR ‘brain bleed*’:ti,ab,kw | 2321 |
| #15 | #13 OR #14 | 193013 |
| #16 | ‘brain hemorrhage’/exp | 192478 |
| #17 | ‘intracranial haemorhag*’:ti,ab,kw OR ‘intracranial hemorthag*’:ti,ab,kw OR 'subarachnoid haemorthag*’:ti,ab,kw OR ‘subarachnoid hemorrhag*’:ti,ab,kw OR ‘intracerebral haemorrhag*’:ti,ab,kw OR ‘intracerebral hemorhag*’:ti,ab,kw OR ‘subdural haemorrhage*’:ti,ab,kw OR ‘subdural hemorrhag*’:ti,ab,kw OR ‘extradural haemorrhage*’:ti,ab,kw OR 'extradural hemorrhag*’:ti,ab,kw | 97087 |
| #18 | #16 OR #17 | 205541 |
| #19 | ‘hemiplegia’/exp | 24644 |
| #20 | hemiplegia*:ti,ab,kw OR hemiparesis*:ti,ab,kw OR paresis*:ti,ab,kw | 48723 |
| #21 | #19 OR #20 | 61818 |
| #22 | #6 OR #9 OR #12 OR #15 OR #18 OR #21 | 906021 |
| #23 | 'cognition'/exp | 3209519 |
| #24 | 'cognitive disorder':ti,ab,kw OR 'cognitive disruption':ti,ab,kw OR 'cognitive impair’:ti,ab,kw OR confusion:ti,ab,kw OR 'neurobehavioral manifestation':ti,ab,kw OR 'neurobehavioral disorder’:ti,ab,kw OR 'cognitive ability':ti,ab,kw OR 'neurobehavioral disruption':ti,ab,kw | 220900 |
| #25 | #23 OR #24 | 3333358 |
| #26 | 'executive function'/exp | 106507 |
| #27 | 'executive dysfunction':ti,ab,kw OR 'dysexecutive syndrome':ti,ab,kw OR 'dysexecutive function':ti,ab,kw OR 'concept formation':ti,ab,kw OR 'goal management':ti,ab,kw OR 'cognitive flexibility":ti,ab,kw OR ‘inhibition control’:ti,ab,kw OR 'working memory':ti,ab,kw | 65907 |
| #28 | #26 OR #27 | 126631 |
| #29 | 'attention'/exp | 358493 |
| #30 | concentration:ti,ab,kw OR vigilance:ti,ab,kw OR inattention:ti,ab,kw OR distract:ti,ab,kw OR awareness:ti,ab,kw OR ‘divided attention’:ti,ab,kw OR ‘focused attention’:ti,ab,kw OR ‘selective attention’:ti,ab,kw OR ‘sustained attention’:ti,ab,kw OR ‘visual attention’:ti,ab,kw OR ‘attention span’:ti,ab,kw | 2152888 |
| #31 | #29 OR #30 | 2354198 |
| #32 | 'memory'/exp | 381323 |
| #33 | forgetting:ti,ab,kw OR ‘episodic memory’:ti,ab,kw OR ‘explicit memory’:ti,ab,kw OR ‘implicit memory’:ti,ab,kw OR ‘long term memory’:ti,ab,kw OR ‘short term memory’:ti,ab,kw OR ‘cognitive aging’:ti,ab,kw OR ‘memory training’:ti,ab,kw | 59961 |
| #34 | #32 OR #33 | 395874 |
| #35 | 'perception disorder'/exp | 45716 |
| #36 | ‘visual construct’:ti,ab,kw OR agnosia:ti,ab,kw OR prosopagnosia:ti,ab,kw OR stereognosis:ti,ab,kw OR ‘auditory perception’:ti,ab,kw OR ‘perceptual distorion’:ti,ab,kw OR ‘perceptual motor processes’:ti,ab,kw OR ‘time perception’:ti,ab,kw OR ‘visual perception’:ti,ab,kw OR ‘perceptual disturbances’:ti,ab,kw OR ‘sensory integration dysfunction’:ti,ab,kw OR hemineglect:ti,ab,kw OR ‘hemi-neglect’:ti,ab,kw OR ‘unilateral neglect’:ti,ab,kw OR ‘spatial neglect’:ti,ab,kw OR ‘spatial-neglect’:ti,ab,kw OR ‘hemi-attention’:ti,ab,kw OR ‘hemi attention’:ti,ab,kw OR visuospatial:ti,ab,kw OR ‘receptive fields’:ti,ab,kw OR ‘sensory neglect’:ti,ab,kw | 52335 |
| #37 | #35 OR #36 | 92337 |
| #38 | #25 OR #28 OR #31 OR #34 OR #37 | 5274640 |
| #39 | #3 AND #22 AND #38 | 151 |

| Web of science | | Total |
| --- | --- | --- |
| #1 | TS=((((((((("Stroke") OR (poststroke OR post-stroke OR "cerebrovascular disorder" OR cerebrovascular OR "cerebral vascular" OR "cerebrovascular disease" OR "basal ganglia cerebral vascular disease" OR CVA OR "cerebrovascular accident" )) OR (("Brain Ischemia") OR ("ischemic encephalopathy" OR "cerebral ischemia" OR "carotid artery disease" OR "intracranial arterial disease" ))) OR (("Brain Infarction") OR ("brain infarct*" OR "cerebral infarct*" OR "cerebral thrombo*" OR "cerebral emboli*" ))) OR (("Cerebral Hemorrhage") OR ("Cerebral haemorrhag*" OR "brain bleed*" ))) OR (( "Intracranial Hemorrhages") OR ("intracranial haemorrhag*" OR "intracranial hemorrhag*" OR "subarachnoid haemorrhag*" OR "subarachnoid hemorrhag*" OR "intracerebral haemorrhag*" OR "intracerebral hemorrhag*" OR "subdural haemorrhage*" OR "subdural hemorrhag*" OR "extradural haemorrhage*" OR "extradural hemorrhag*" ))) OR (("Hemiplegia") OR (hemiplegia* OR hemiparesis* OR paresis* ))))) and Preprint Citation Index (Exclude – Database) | 1367705 |
| #2 | TS=(((((((("Perceptual Disorders") OR ("visual perception" OR "visual construct" OR agnosia OR prosopagnosia OR stereognostic OR "auditory perception" OR "perceptual distortion" OR "perceptual motor processes" OR "time perception" OR "visual perception" OR "perceptual disturbances" OR "sensory integration dysfunction" OR hemineglect OR "hemi-neglect" OR "unilateral neglect" OR "spatial neglect" OR "spatial-neglect" OR "hemi-attention" OR "hemi attention" OR visuospatial OR "receptive fields" OR "sensory neglect" )) OR (("Memory") OR (forgetting OR "episodic memory" OR "explicit memory" OR "implicit memory" OR "long term memory" OR "short term memory" OR "cognitive aging" OR "memory training" ))) OR (("Attention") OR (concentration OR vigilance OR inattention OR distract* OR awareness OR "divided attention" OR "focused attention" OR "selective attention" OR "sustained attention" OR "visual attention" OR "attention span" ))) OR (("Executive Function") OR ("executive dysfunction" OR "dysexecutive syndrome" OR "dysexecutive function" OR "concept formation" OR "goal management" OR "cognitive flexibility" OR "inhibition control" OR "working memory" ))) OR (("Cognition") OR ("cognitive disorder" OR "cognitive disruption" OR "cognitive impair*" OR confusion OR "neurobehavioral manifestation" OR "neurobehavioral disorder" OR "cognitive ability" OR "neurobehavioral disruption" ))))) and Preprint Citation Index (Exclude – Database) | 13052479 |
| #3 | TS=(("action observation" OR "action observation training" OR "action observation treatment" OR "action observation therapy" OR "action observation-execution" OR "motor observation" OR "movement observation" OR "motion observation" OR "gesture observation" )) and Preprint Citation Index (Exclude – Database) | 4354 |
| #4 | #1 AND #2 AND #3 | 117 |

| EBSCO | | Total |
| --- | --- | --- |
| #1 | SU=(((((((("Stroke") OR (poststroke OR post-stroke OR "cerebrovascular disorder" OR cerebrovascular OR "cerebral vascular" OR "cerebrovascular disease" OR "basal ganglia cerebral vascular disease" OR CVA OR "cerebrovascular accident" )) OR (("Brain Ischemia") OR ("ischemic encephalopathy" OR "cerebral ischemia" OR "carotid artery disease" OR "intracranial arterial disease" ))) OR (("Brain Infarction") OR ("brain infarct*" OR "cerebral infarct*" OR "cerebral thrombo*" OR "cerebral emboli*" ))) OR (("Cerebral Hemorrhage") OR ("Cerebral haemorrhag*" OR "brain bleed*" ))) OR (( "Intracranial Hemorrhages") OR ("intracranial haemorrhag*" OR "intracranial hemorrhag*" OR "subarachnoid haemorrhag*" OR "subarachnoid hemorrhag*" OR "intracerebral haemorrhag*" OR "intracerebral hemorrhag*" OR "subdural haemorrhage*" OR "subdural hemorrhag*" OR "extradural haemorrhage*" OR "extradural hemorrhag*" ))) OR (("Hemiplegia") OR (hemiplegia* OR hemiparesis* OR paresis* )))) | 1248714 |
| #2 | SU=((((((("Perceptual Disorders") OR ("visual perception" OR "visual construct" OR agnosia OR prosopagnosia OR stereognostic OR "auditory perception" OR "perceptual distortion" OR "perceptual motor processes" OR "time perception" OR "visual perception" OR "perceptual disturbances" OR "sensory integration dysfunction" OR hemineglect OR "hemi-neglect" OR "unilateral neglect" OR "spatial neglect" OR "spatial-neglect" OR "hemi-attention" OR "hemi attention" OR visuospatial OR "receptive fields" OR "sensory neglect" )) OR (("Memory") OR (forgetting OR "episodic memory" OR "explicit memory" OR "implicit memory" OR "long term memory" OR "short term memory" OR "cognitive aging" OR "memory training" ))) OR (("Attention") OR (concentration OR vigilance OR inattention OR distract* OR awareness OR "divided attention" OR "focused attention" OR "selective attention" OR "sustained attention" OR "visual attention" OR "attention span" ))) OR (("Executive Function") OR ("executive dysfunction" OR "dysexecutive syndrome" OR "dysexecutive function" OR "concept formation" OR "goal management" OR "cognitive flexibility" OR "inhibition control" OR "working memory" ))) OR (("Cognition") OR ("cognitive disorder" OR "cognitive disruption" OR "cognitive impair*" OR confusion OR "neurobehavioral manifestation" OR "neurobehavioral disorder" OR "cognitive ability" OR "neurobehavioral disruption" )))) | 3876624 |
| #3 | SU=("action observation" OR "action observation training" OR "action observation treatment" OR "action observation therapy" OR "action observation-execution" OR "motor observation" OR "movement observation" OR "motion observation" OR "gesture observation" ) | 2112 |
| #4 | #1 AND #2 AND #3 | 11 |

| CNKI | | Total |
| --- | --- | --- |
| #1 | (TKA=(脑血管病 + 脑卒中 + 脑梗塞 + 脑出血 + 缺血性卒中 + 出血性卒中 + 偏瘫)) AND (TKA=(动作观察疗法 + 动作观察训练)) AND (TKA=(认知功能 + 神经行为障碍 + 视空间能力 + 认知处理速度 + 执行力 + 注意力 + 意识 + 记忆力 + 遗忘 + 听觉知觉障碍 + 单侧忽略 + 感知障碍)) | 12 |

| VIP | | Total |
| --- | --- | --- |
| #1 | M=(脑血管病 OR 脑卒中 OR 脑梗塞 OR 脑出血 OR缺血性卒中 OR出血性卒中OR偏瘫)OR =(脑血管病 OR 脑卒中 OR 脑梗塞 OR 脑出血 OR缺血性卒中OR出血性卒中 OR 偏瘫)AND (M=(动作观察疗法 OR动作观察训练) OR =(动作观察疗法 OR动作观察训练)AND(M=(认知功能 OR神经行为障碍 OR视空间能力 OR认知处理速度 OR 执行力 OR 注意力 OR 意识 OR记忆力 OR 遗忘 OR听觉知觉障碍 OR 单侧忽路 OR感知障碍) OR =(认知功能 OR 神经行为障碍 OR视空间能力 OR认知处理速度 OR 执行力 OR 注意力 OR 意识 OR 记忆力 OR遗忘 OR 听觉知觉障碍 OR 单侧忽略 OR 感知障碍)) | 8 |

| Wanfang | | Total |
| --- | --- | --- |
| #1 | 主题:(脑血管病 or 脑卒中 or 脑梗塞 or 脑出血 or 缺血性卒中 or 出血性卒中 or 偏擁) and 主题:(动作观察疗法 or 动作观察训练) and 主题:(认知功能 or 神经行为障碍 or 视空间能力 or 认知处理速度 or 执行力 or 注意力 or 意识 or 记忆力 or 遗忘 or 听觉知觉障碍 or 单侧忽略 or 感知障碍) | 70 |
